# Heat Stress Modulates a Placental Immune Response Associated With Alterations in the Development of the Fetal Intestine and Its Innate Immune System in Late Pregnant Mouse

**DOI:** 10.3389/fphys.2022.841149

**Published:** 2022-04-04

**Authors:** Huiduo Guo, Riliang Liu, Jianwen He, Wen Yao, Weijiang Zheng

**Affiliations:** ^1^ College of Animal Science and Technology, Nanjing Agricultural University, Nanjing, China; ^2^ College of Biotechnology, Jiangsu University of Science and Technology, Zhenjiang, China; ^3^ Clinical Research Center, Affiliated Hospital of Shaanxi University of Chinese Medicine, Shaanxi University of Chinese Medicine, Xianyang, China; ^4^ Key Lab of Animal Physiology and Biochemistry, Ministry of Agriculture and Rural Affairs of the People’s Republic of China, Nanjing Agricultural University, Nanjing, China

**Keywords:** heat stress, late gestation, intestinal development, placenta, mouse

## Abstract

The placenta is critical for the regulation of fetal innate immune function. Maternal heat stress (HS) impairs the immune function and the intestinal barrier in the offspring. However, the effects of maternal HS on the placental immune response and the development of the fetal intestine and its innate immune system remain unclear. Fetal mice were divided into the utero control (IUTN) and heat stress (IUHS) groups according to the maternal ambient temperature. Transcriptome analysis revealed that the expressions of placental immune response–related genes such as macrophage antigen CD68 and Fc gamma receptors 1 and 3 (*fcgγ1* and *fcgγ3*) were increased, but the mRNA expression and protein levels of colony-stimulating factor-1 (Csf1) were decreased in the HS group compared with the TN group (*p* < 0.05). Furthermore, there was no significant difference in the intestinal length normalized to pup weight between the IUTN and IUHS groups. The expression of genes (such as *alpi* and *ttr*) involved in fetal duodenum and jejunum development was downregulated by maternal HS, whereas the expression of genes enriched in the cell cycle was increased. The mRNA expression and protein levels of cell division cycle 6 (Cdc6) in the fetal duodenum and jejunum were much higher in the IUHS group than in the IUTN group (*p* < 0.05). Maternal HS also down-regulated the expression of genes enriched in the innate immune system in the fetal duodenum and jejunum. The mRNA expression and protein levels of interleukin 1 alpha (IL1a) were reduced in the IUHS group compared with the IUTN group (*p* < 0.05). Taken together, these data demonstrated that maternal HS modulated the expression of genes in the placenta related to the immune response and inhibited the development of the fetal intestine and its innate immune system.

## Introduction

With the rising global temperatures, heat stress (HS) induced by high ambient temperature threatens animal health performance (Belhadj Slimen et al., 2016). HS during late gestation is usually linked with the poor reproductive performance (such as a reduction in birth weight, average daily weight gain, and brain weight), alters the carcass composition, and increases the morbidity and mortality in the offspring (Johnson et al., 2015; Laporta et al., 2017). Changing the intrauterine environment conditions impairs the structure and function of the offspring’s organs, increasing the risk of various chronic diseases (such as abnormal growth, metabolic disorders, and immune dysfunction), which commonly occur in their postnatal life (Reynolds et al., 2019). Late gestation is a crucial period of intestinal development. It has been reported that the development of the fetal intestine begins in early gestation and accelerates during late gestation, and the gut is sensitive to the alterations of the early life environment, especially heat stress (Trahair and Sangild, 1997). The development of intestinal innate immune system is influenced by a combination of internal processes (genetic and endocrine) and environmental factors (such as the amniotic fluid, colostrum, and microorganisms) (Pan et al., 2020). Our previous studies showed that HS during late gestation increased the serum adrenocorticotropic hormone of the newborn piglets (Guo et al., 2020) and the brain corticosterone levels in the fetal mice (Guo et al., 2021). Furthermore, psychological stress in gestational mice stimulates the production of corticosterone in their offspring, contributing to the dysfunction of the offspring’s intestinal barrier and blunting of their epithelial architecture (Shah et al., 2019). However, the consequences of maternal HS during late gestation on the morphological structure and development of the fetal intestine are still not fully understood.

To build a functional and mature immune system, a series of highly coordinated developmental events occur during the embryonic life and continue through the early postnatal period (Holsapple et al., 2003). Several studies have shown that maternal HS reduces the circulating immunoglobulin (IgG) in calves (Tao et al., 2012) and piglets (Machado-Neto et al., 1987), which negatively influenced the transfer of passive immunity (Dahl et al., 2020), and retards the growth and maturation of the immune system in the offspring (Dahl et al., 2020). Stress as a modulator of gut inflammation may induce the attenuation of the hypothalamus pituitary adrenal axis (Glover et al., 2010), thereby changing gut physiology (McElroy and Weitkamp, 2011), which may cause poor intestinal immune development and epithelial injury (Wei et al., 2019). Our previous study also showed that maternal HS increased intestinal permeability of the newborn piglets (Guo et al., 2020). Increased intestinal permeability is usually accompanied by the higher risk of bacterial translocation and systemic inflammation (Lambert, 2004). Furthermore, prenatal stress may impair the development of the offspring’s intestinal tissues, resulting in the intestinal inflammation and necrotizing enterocolitis (Yeramilli et al., 2020). Repeated social stress applied to late pregnant sows has long-lasting effects on several parameters of the offspring’s immune function (Couret et al., 2009). However, whether HS during late gestation affects the development of the fetal intestinal immune system remains to be illuminated.

Most maternal immunoglobulin G (IgG) is acquired in the fetus during the third trimester (Simister, 2003). Acquired IgG is extremely important for the neonate to adapt to the extra-uterine environment (Chucri et al., 2010). In addition, the placenta plays an important role in the transfer of maternal antibodies to the fetus and protects them from harmful substances (Tong and Abrahams, 2020). Early prenatal stress up-regulates the expression of genes involved in the pro-inflammatory response in the placenta, resulting in the hyperactive performance of the male offspring (Bronson and Bale, 2014). Our previous work reported that maternal HS disrupted the function of the placental barrier function, which might impair the function of the fetal brain and intestine (Guo et al., 2021). However, whether HS during late gestation affects the placental immune response and its relationship with fetal intestinal development and innate immune function remains to be investigated.

To better understand the interactions between maternal HS and the intestinal development in the offspring, we employed RNA-seq to determine the genes expression changes in the placenta and fetal duodenum and jejunum in a mouse model. Our data showed that maternal HS decreased the expression of genes associated with the fetal intestinal development, possibly due to the increase of the expression of genes enriched in the cell cycle. Maternal HS also inhibited the expression of genes related to the innate immune system in the fetal intestine which might be associated with the expression of genes involved in a placental inflammatory response.

## Materials and Methods

### Animals

All experiments were approved by the Animal Care and Use Committee of Nanjing Agricultural University (Approval number: N1822223), China. The ICR strains of female and male (6–8 weeks old) mice were obtained from the Model Animal Research Center of Nanjing University, Nanjing, China. All animals were housed in a 12:12 h light: dark cycle and allowed *ad libitum* access to the food and water. One male and two female mice were kept in each cage for successful mating, and once pregnancy was determined via a vaginal plug, the subsequent morning the pregnant individual was moved into a separate cage. From gestation days 12.5 to 18.5, the pregnant mice were randomized to the two environmental conditions as previously reported (He et al., 2021): the control (TN) group mice, which were fed at room temperature (24 ± 1°C), and the HS group mice, which were kept in the artificial intelligence climate chamber (35 ± 1°C).

### Placenta and Fetal Gut Collection

On gestation day 18.5, all pregnant mice were sacrificed by cervical dislocation after carbon dioxide anesthesia. According to the maternal temperatures, the fetal mice were also divided into the utero control (IUTN) and heat stress (IUHS) groups. The fetal mice were immediately removed from the dams in the TN and HS groups, respectively. The placenta and fetal intestine were collected and then stored at −80°C until use.

### Morphological Analysis of Fetal Intestine

The fetal duodenum, jejunum, and ileum were fixed in 4% paraformaldehyde and paraffin-embedded, and sections were cut at 5 µM thickness with a rotary microtome (Leica RM2235, Germany). The fetal small intestine was stained with the hematoxylin and eosin (HE) kit (Solarbio, China) for morphological analysis according to the manufacturer’s instructions. Photomicrographs were taken with a virtual microscope (Olympus, Tokyo, Japan). The villus height (from the tip of the villus to the villus–crypt junction) in the fetal duodenum, jejunum, and ileum was measured using the ImageJ software (Rawak Software Inc., Stuttgart, Germany). All the raw and processed data were deposited in the Gene Expression Omnibus database (GSE192968; https://www.ncbi.nlm.nih.gov/geo/info/linking.html).

### RNA Extraction and Transcriptome Sequencing in Placenta, Fetal Duodenum and Jejunum

The RNAiso Plus reagent (Takara, Tokyo, Japan) was used to extract total RNA from the whole placenta and fetal duodenum and jejunum homogenates according to the manufacturer’s protocol as previously reported (Guo et al., 2021). RNA integrity was determined using Bioanalyzer 2200 (Agilent Technologies, Santa Clara, CA, United States). Only the samples with RNA integrity number scores > 8.0 were used for cDNA library construction.

The cDNA library construction and RNA‐seq analysis were conducted at the Shanghai NovelBio Bioinformatics Company. The adapters and low-quality bases were removed to obtain the clean data. High-quality clean reads obtained from quality control were mapped to the *Mus musculus* reference genome (GRCm38.p5) by the Hisat2 aligner and kept for further downstream sequence analysis.

### Analysis of Differentially Expressed Genes

The raw digital gene expression counts were calculated based on the fragments per kilobase of exon per million fragments (FPKM). Analyses, based on differentially expressed genes (DEGs), the placenta (two biological duplications in each group), and the fetal duodenum and jejunum (three biological duplications in each group), were performed based on the DESeq2 method. The fold change (FC) > 1.5 or < −1.5, *p* < 0.05, and FDR < 0.05 were used as significance thresholds.

### Interaction Networks of Differentially Expressed Genes and Hub Genes Analysis

To investigate the relationships between the proteins and DEGs identified in this study, protein–protein interaction (PPI) networks of DEGs were obtained using the STRING database. The networks were visualized in Cytoscape (version 3.6.0). Subnets (molecular complex detection (MCODE) score ≥9 in the placenta, fetal duodenum and jejunum) were selected using the MCODE plugin in Cytoscape (version 3.6.0). Additionally, hub genes in the PPI network module were obtained using the CytoHubba plugin in Cytoscape (version 3.6.0). Finally, the DEGs of the subnets and hub genes were used for GO functional enrichment analysis.

### Quantitative Real-Time PCR Analysis of Differentially Expressed Genes

The mRNA of DEGs was validated by relative quantitative real-time PCR (qRT-PCR). The elimination of genomic DNA contamination and isolated RNA were reverse transcribed into cDNA using a PrimeScript RT Reagent Kit with gDNA Eraser (Takara, Japan). qRT-PCR was performed with TB Green qPCR Master Mix (Takara) on an RT-PCR detection system (Thermo Fisher Scientific). The sequences of primers were designed using the NCBI tool and are listed in Table 1. The following PCR cycling conditions were used for each assay: enzyme activation at 95°C for 15 s, amplification of the gene product through 40 successive cycles of 95°C for 5 s and then 60°C for 30 s, and generation of the dissolution curve of the gene at 95°C for 15 s, then 60°C for 30 s, and then 95°C for 15 s. In the present study, *gapdh* (glyceraldehyde-3-phosphate dehydrogenase) was identified as the reference gene. Relative quantification of target mRNAs was calculated and normalized to *gapdh* expression using the 2^−ΔΔCt^ method.

**TABLE 1:** Sequence of primers used in RT-PCR.

Target gene	Primer sequence (5′ to 3′)	Gene bank access
bub1b	F: CAG​TCC​CAG​CAC​AGA​CAG​TT	NM_009773.3
	R: GAC​GCG​GTA​TCG​GCA​TTT​TC	
bub1	F: AGC​ATG​AGC​AGT​GGG​TTA​GT	NM_009772.2
	R: TTC​CTG​CTG​GGA​GCA​AGT​AT	
cdc6	F: TCT​GCA​AGA​CTT​CAA​GAA​GGA​AG	NM_011799.2
	R: ACG​ATC​ATG​GGG​CCT​TTC​TC	
cdc7	F: ACT​TCT​GTG​AAC​CCT​GCT​GC	NM_009863.3
	R: AAA​GAG​TCA​TCA​GCC​GGA​CA	
il1α	F: AGG​GAG​TCA​ACT​CAT​TGG​CG	NM_010554.4
	R: CTT​CCC​GTT​GCT​TGA​CGT​TG	
ifit3	F: TGT​GGA​GTG​CTG​CTT​ATG​GG	NM_010501.2
	R: TCA​AAA​GGT​GCT​CTG​TCT​GC	
ptx3	F: CGT​GCA​TCC​TGT​GAG​ACC​AA	NM_008987.3
	R: ACC​AAC​ACT​AGG​GAC​TGG​GA	
ptgs2	F: CAT​CCC​CTT​CCT​GCG​AAG​TT	NM_011198.4
	R: CAT​GGG​AGT​TGG​GCA​GTC​AT	
cd68	F: TGT​TCA​GCT​CCA​AGC​CCA​AA	NM_001291058.1
	R: ACT​CGG​GCT​CTG​ATG​TAG​GT	
csf1	F: GGG​CCT​CCT​GTT​CTA​CAA​GT	NM_001113530.1
	R: TGG​TGA​GGG​GTC​ATA​GAA​TCC	
gapdh	F: GGC​TCC​CTA​GGC​CCC​TCC​TG	XM_036165840.1
	R: TCC​CAA​CTC​GGC​CCC​CAA​CA	

### Western Blot Analysis

The remaining placenta and fetal duodenum and jejunum samples were homogenized at 4°C in 50 mmol/L Tris-HCl buffer (pH 7.4) containing 150 mmol/L NaCl, 1% NP-40, 0.5% sodium deoxycholate, 0.1% sodium dodecyl sulfate (SDS), and protease inhibitor cocktail (Beyotime Biotechnology, China) by using a bead cell disrupter (Tomy, Japan). Protein concentrations were measured with a BCA protein assay kit (Thermo Fisher, United States). Total tissue proteins (20–40 μg) were used for electrophoresis on an SDS-15, -12, or -10% polyacrylamide gel and then transferred to a polyvinylidene fluoride membrane. Western blot analysis was performed according to manual instructions provided by the primary antibody suppliers. Polyclonal antibodies against Cdc6 (1:1,000), Il1α (1:1,000), Cd68 (1:1,000), Csf1 (1:1,000), and β-actin and gapdh (1:10,000) were selected as a loading control. All antibodies were obtained from Proteintech (Wuhan, China) and Huabio (Hangzhou, China).

### Statistical Analysis

Statistical analyses were carried out by using SPSS 19.0 (IBM SPSS, Chicago, IL, United States), except for the RNA-seq results. Data were presented as mean ± SEM. *p* < 0.05 was considered statistically significant. The difference of each indicator between the two groups was analyzed using the independent-sample *t*-tests.

## Results

### The Expression of Placental Immune Response–Related Genes Was Activated by Maternal HS

**FIGURE 1:**
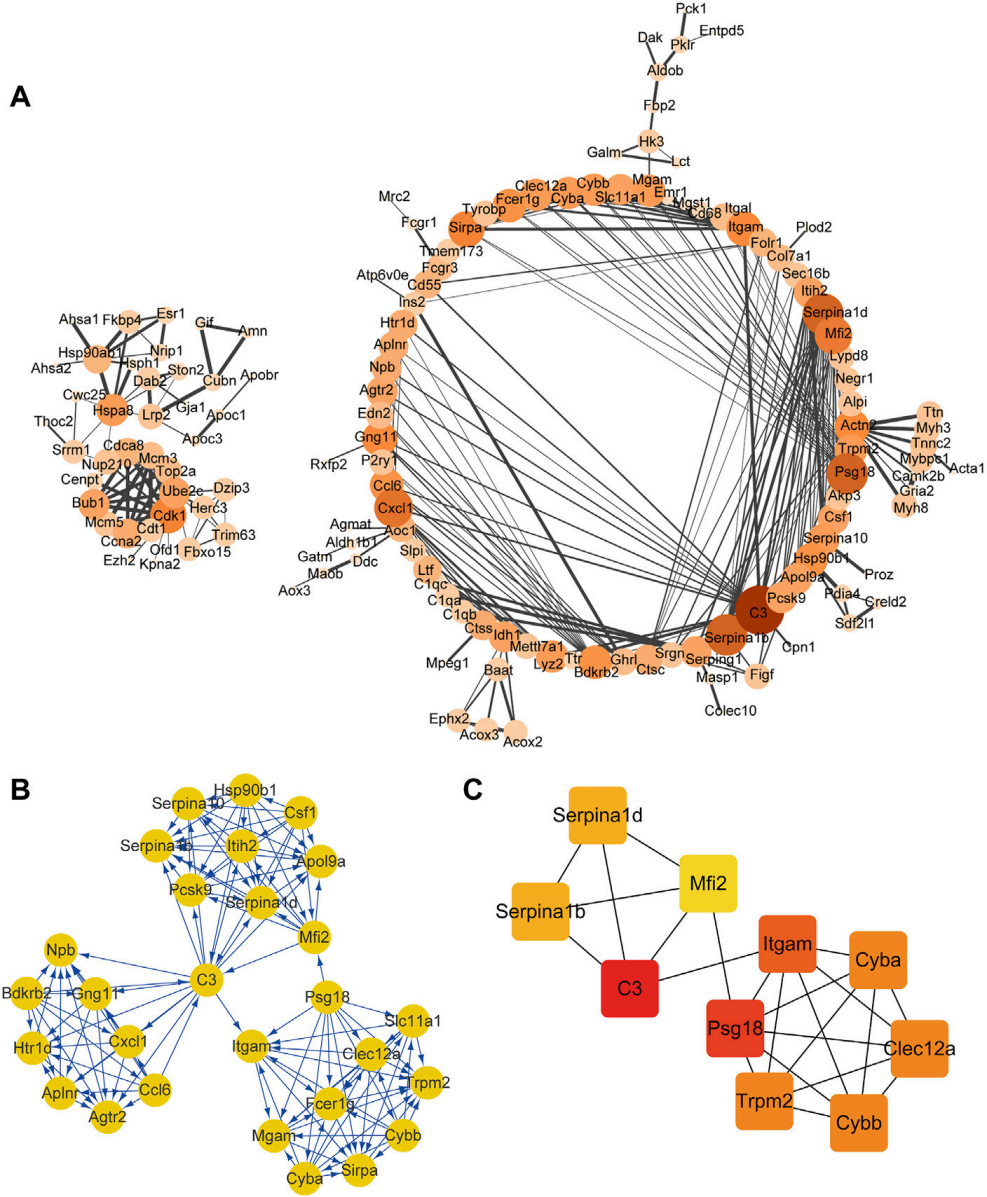
PPI network (confidence score ≥9) and hub gene analysis in the placenta. PPI network analysis **(A)**, MCODE cluster of PPI networks **(B)**, and the top 10 hub genes **(C)** in the placenta. The color of the node represents the clustering coefficient. The size of each node is proportional to the number of degrees. Interactions are indicated by edges, with thicker edges having stronger associations. Different colors represent the different importance of the hub genes, in terms of their degree of connectivity. Higher ranks are represented by a deeper color.

A total of 604 nodes and 432 edges were contained in the PPI network (confidence score ≥0.9; Figure 1A). One module (MCODE score ≥9) was obtained from the PPI network constructed using the MCODE plugin in Cytoscape, and these DEGs of the subnet were mainly enriched in the inflammatory response and response to cytokines in the placenta (Figure 1B). Using the CytoHubba plugin in Cytoscape, the top ranked 10 hub genes were mainly related to the inflammatory response and were listed as follows: complement C3 (*c3*), pregnancy-specific beta 1 glycoprotein (*psg18*), integrin subunit alpha M (*itgam*), cytochrome b-245 alpha chain (*cyba*), cytochrome b-245 beta chain (*cybb*), C-type lectin domain family 12 member A (*clec12a*), transient receptor potential cation channel subfamily M member 2 (*trpm2*), serpin family A member 1b (*serpina1b*), serpin family A member 1d (*serpina1d*), and melanotransferrin 2 (*mfi2*) (Figure 1C).

### Maternal HS Increased the Expression of Genes Associated With Placental Inflammatory Response

**FIGURE 2:**
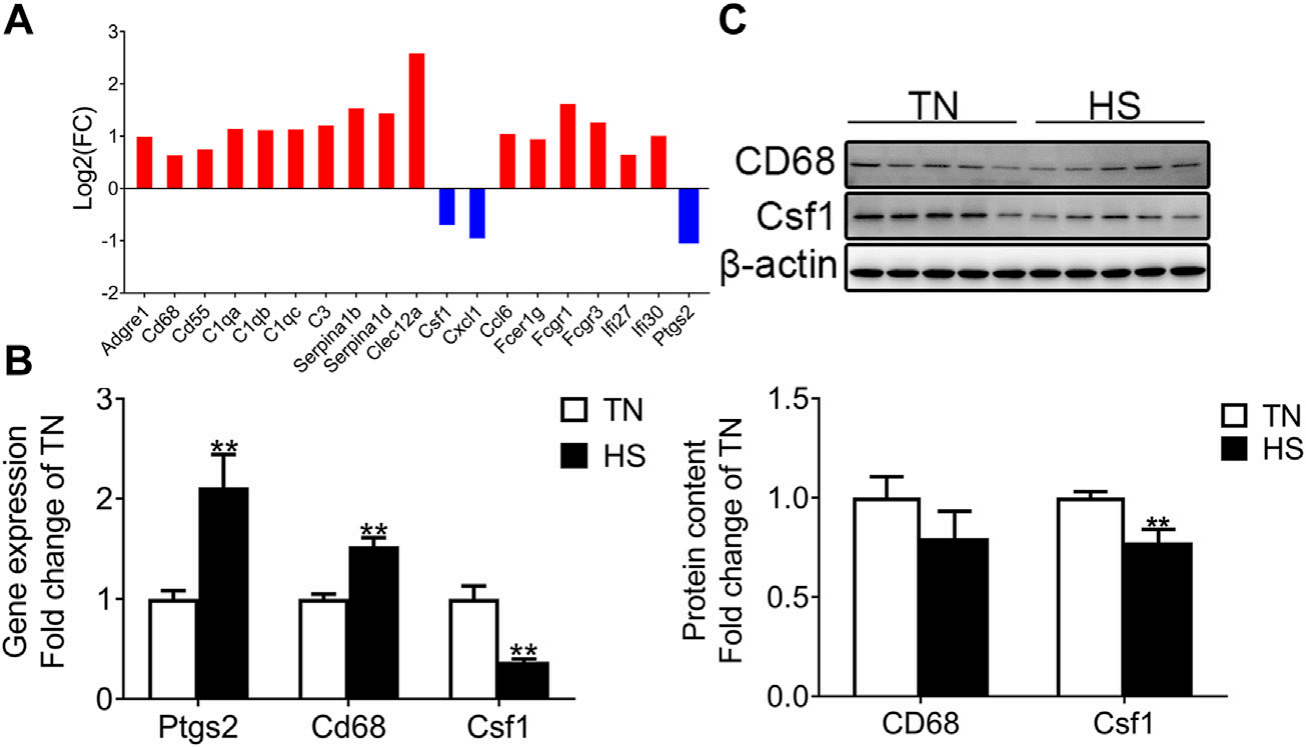
Effects of HS during late gestation on the expression of genes enriched in immune response in the placenta. Fold change of DEGs **(A)**, mRNA expression **(B)**, and protein levels (TN: *n* = 11; HS: *n* = 8) **(C)** in the placenta. Gene symbols are shown on the x-axis, and log_2_ FC is shown on the y-axis. Blue bar: down-regulation; red bar: up-regulation. Data are presented as mean ± SEM. *p < 0.05, **p < 0.01.

The interested DEGs associated with the inflammatory responses in the placenta were randomly chosen and analyzed. As shown in Figure 2A, maternal HS increased the expression of inflammatory response–related genes [adhesion G protein-coupled receptor E1 (*adgre1*), CD68 molecule (*cd68*), CD55 molecule (*cd55*), complement c1q A chain (*c1qa*), complement c1q B chain (*c1qb*), complement c1q C chain (*c1qc*), *c3*, *serpina1b*, *serpina1d*, *clec12a*, C-C motif chemokine ligand 6 (*ccl6*), Fc fragment of IgE receptor Ig (*fcer1g*), Fc fragment of IgG receptor Ia (*fcgr1*), Fc fragment of IgG receptor IIIa (*fcgr3*), interferon alpha inducible protein 27 (*ifi27*), and interferon alpha inducible protein 30 (*ifi30*)] and decreased the expression of colony-stimulating factor-1 (*csf1*), C-X-C motif chemokine ligand 1 (*cxcl1*), and prostaglandin-endoperoxide synthase 2 (*ptgs2*) compared with the IUTN group. To validate the reliability of RNA-seq, qRT-PCR was applied to determine the mRNA expression of some related genes, such as cd68, *ptgs2*, and *csf1*. The mRNA expression of *cd68* and *ptgs2* were up-regulated by maternal HS, while the mRNA expression of *csf1* was down-regulated in the IUHS group compared with the IUTN group (*p* < 0.05) (Figure 2B). There was no significant difference in the protein level of placental Cd68 between the TN and HS groups. However, the protein levels of Csf1 were reduced in the HS group (Figure 2C) compared with the TN group (*p* < 0.05).

### Maternal HS Decreased the Intestinal Length and Villus Height of Fetal Mice

**FIGURE 3:**
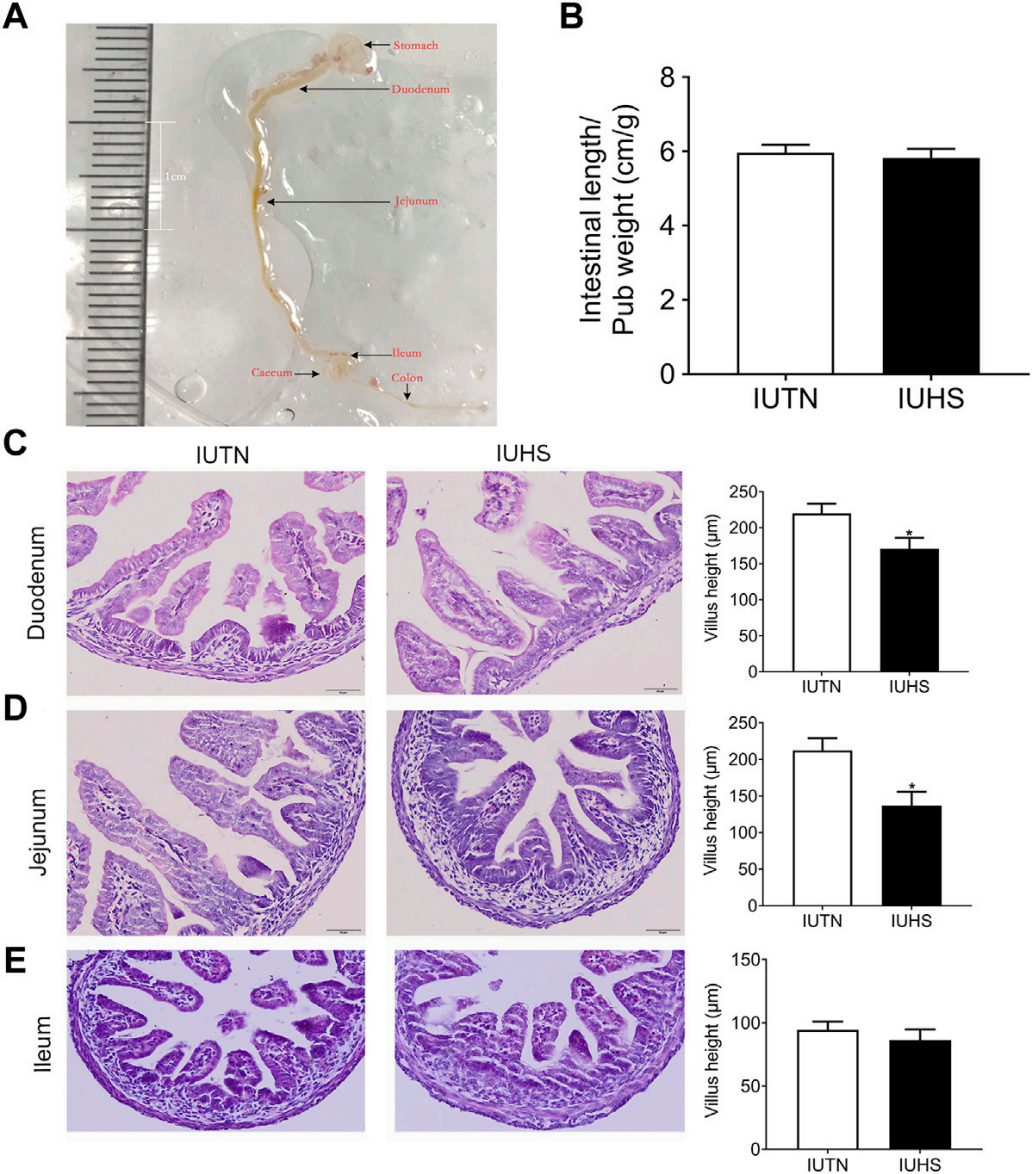
Effects of maternal heat stress on the length and morphology of the fetal intestine. **(A)** Anatomical structure of the fetal intestine; **(B)** length of the fetal intestine normalized to pub weight (IUTN, *n* = 11; IUHS, *n* = 8); **(C–E)** HE histology (left) and quantification of villus height (right) in the fetal duodenum **(C)**, jejunum **(D)**, and ileum **(E)**. IUTN: *in utero* TN group; IUHS: *in utero* HS group; *n* = 7. Scale bar: 100 μM. Data are presented as mean ± SEM. *p < 0.05.

Our previous study showed that a similar number of live fetuses were in the TN and HS groups (TN 12.82 ± 0.81, HS 13.13 ± 0.72), and the pub weight had no significant difference in the two groups (TN 1.00 ± 0.05 g, HS 0.98 ± 0.07) (Guo et al., 2021). The anatomy of the small intestine is shown in Figure 3A. As shown in Figure 3B, the intestinal length normalized to pub weight in the IUHS group was not different in the IUTN group (*p* < 0.05). Representative morphologic observations of intestine tissue in the fetal duodenum, jejunum, and ileum from the IUTN and IUHS groups were shown in Figure 3. Morphological examination revealed that the villus height of the fetal duodenum (Figure 3C) and jejunum (Figure 3D) (*p* < 0.05) was reduced by maternal HS. However, there was no significant difference in the villus height of fetal ileum between the IUTN and IUHS groups (*p* > 0.05) (Figure 3E).

### Maternal HS Inhibited the Expression of Genes Related to Intestinal Development in the Fetal Mice

The expressions of genes related to intestinal development in the fetal duodenum and jejunum were listed in Tables 2, 3, respectively. For the fetal duodenum, the expressions of enterocyte-related markers [including alkaline phosphatase, intestinal (*alpi*), apolipoprotein C3 (*apoc3*), apolipoprotein A4 (*apoa4*), ATP-binding cassette subfamily G member 5 (*abcg5*), ATP-binding cassette subfamily G member 8 (*abcg8*), solute carrier family 2 member 2 (*slc2a2*), neuraminidase 1 (*neu1*), and transthyretin (*ttr*)] were down-regulated in the IUHS group compared with the IUTN group. Maternal HS also reduced the expression of intestinal epithelial cell markers [fatty-acid–binding protein 2 (*fabp2*) and microsomal triglyceride transfer protein (*mttp*)] and enteroendocrine cell marker [gastric inhibitory polypeptide (*gip*)] (Table 2).

**TABLE 2:** Markers of intestinal development in the fetal duodenum between IUTN and IUHS groups.

Gene	Description	GO term	Fold change (log_2_)
alpi	Alkaline phosphatase	GO:0008152 metabolic process	−1.6
apoc3	Apolipoprotein C-III	GO:0042953 lipoprotein transport	−1.8
apoa4	Apolipoprotein A-IV	GO:0042157 lipoprotein metabolic process	−1.9
abcg5	ATP-binding cassette subfamily G member 5	GO:0042632 cholesterol homeostasis	−2.4
		GO:0006810 transport	
abcg8	ATP-binding cassette subfamily G member 8	GO:0042632 cholesterol homeostasis	−2.8
slc2a2	Solute carrier family 2 facilitated glucose transporter member 2	GO:0031018 endocrine pancreas development	−1.9
neu1	Sialidase-1	GO:0005975 carbohydrate metabolic process	−2.1
ttr	Transthyretin	GO:0070327 thyroid hormone transport	−1.6
fabp2	Fatty-acid–binding protein, intestinal	GO:0015909 long-chain fatty acid transport	−1.9
mttp	Microsomal triglyceride transfer protein large subunit	GO:0042157 lipoprotein metabolic process	−1.5
gip	Gastric inhibitory polypeptide	GO:0042594 response to starvation	−1.7

Positive represents up-regulated; negative represents down-regulated.

**TABLE 3:** Markers of intestinal development in the jejunum between the IUTN and IUHS groups.

Gene	Description	GO term	Fold change (log_2_)
alpi	Alkaline phosphatase	GO:0008152 metabolic process	−1.5
apoa4	Apolipoprotein A-IV	GO:0042157 lipoprotein metabolic process	−1.7
car12	Carbonic anhydrase 12	GO:0008152 metabolic process	−1.6
ttr	Transthyretin	GO:0070327 thyroid hormone transport	−2.8
hes1	Transcription factor HES-1	GO:0061106 negative regulation of stomach neuroendocrine cell differentiation	−1.6
msi1	RNA-binding protein musashi homolog 1	GO:0030855 epithelial cell differentiation	−1.6
dkk1	Dickkopf-related protein 1	GO:1901296 negative regulation of canonical Wnt signaling pathway involved in cardiac muscle cell fate commitment	−3.4
sfrp5	Secreted frizzled-related protein 5	GO:2000057 negative regulation of Wnt signaling pathway involved in digestive tract morphogenesis	5.0

Positive represents up-regulated; negative represents down-regulated.

For the fetal jejunum, the expressions of *alpi*, carbonic anhydrase 12 (*car12*), *ttr*, Hes family BHLH transcription factor 1 (*hes1*), musashi RNA-binding protein 1 (*msi1*), and dickkopf-related protein 1 (*dkk1*) associated with intestinal development were higher in the IUHS group than those in the IUTN group. However, the expression of secreted frizzled-related protein 5 (*sfrp5*) was up-regulated in the IUHS group compared with the IUTN group (Table 3).

### Maternal HS Had Profound Effects on the Cell Cycle of the Fetal Intestine

**FIGURE 4:**
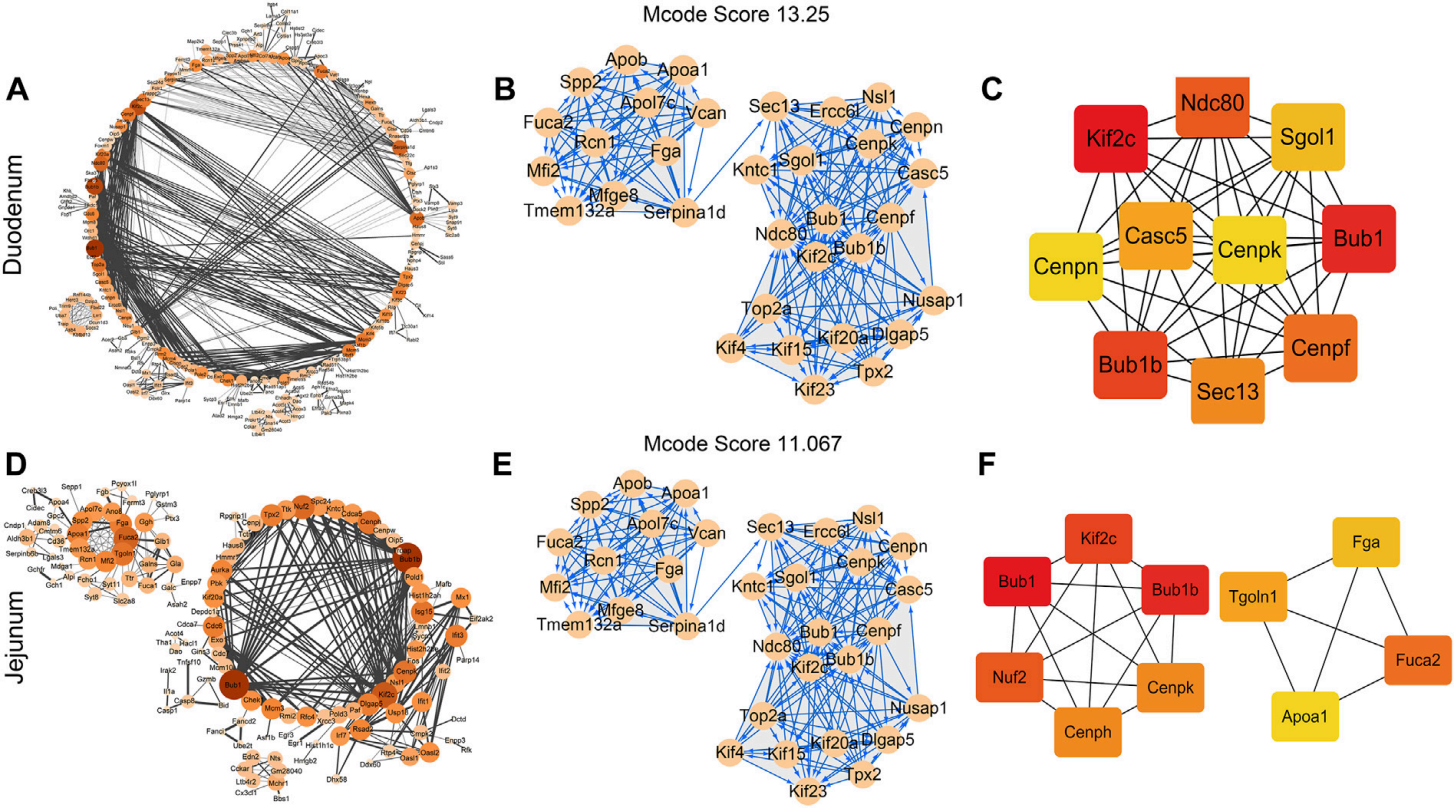
Protein–protein interaction (PPI) network and hub gene analysis of DEGs in the fetal duodenum and jejunum. **(A)** PPI network interaction in the fetal duodenum. Interactions are indicated by edges, with thicker edges having stronger associations. MCODE cluster of PPI networks **(B)** and the top 10 hub genes **(C)** in the fetal duodenum. PPI network interaction **(D)**, MCODE cluster of PPI networks **(E)**, and the top 10 hub genes **(F)** in the fetal jejunum. The color of the node represents the clustering coefficient. The size of each node is proportional to the number of degrees. Interactions are indicated by edges, with thicker edges having stronger associations. Different colors represent the differing importance of the hub genes, in terms of their degree of connectivity. Higher ranks are represented by a deeper color.

The differentially expressed genes (DEGs) (confidence score ≥0.9) in the fetal duodenum and jejunum were used for the PPI network and hub genes analysis. For the fetal duodenum (Figure 4A), a total of 948 nodes (genes) and 887 edges (interactions) were contained in the PPI network. By using the MCODE plugin in Cytoscape, only one subnet was obtained with an MCODE score ≥13, and the DEGs of the subnet were mainly enriched in the cell cycle, cell division, mitotic nuclear division, immune system process, innate immune response, and ATP catabolic process (Figure 4B). The top ranked 10 hub DEGs were chosen by the CytoHubba plugin in Cytoscape and sequentially ordered as follows: kinesin family member 2C (*kif2c*), BUB1 mitotic checkpoint serine/threonine kinase (*bub1*), BUB1 mitotic checkpoint serine/threonine kinase B (*bub1b*), NDC80 kinetochore complex component (*ndc80*), centromere protein F (*cenpf*), SEC13 homolog, nuclear pore and COPII coat complex component (*sec13*), *casc5*, Shugoshin 1 (*sgol1*), centromere protein N (*cenpn*), and centromere protein K (*cenpk*) (Figure 4C). Hub DEGs were mainly associated with the cell cycle, cell division, and mitotic nuclear division.

For the fetal jejunum, it was identified that a total of 640 nodes and 463 edges were involved in the PPI network (Figure 4D). Using the MCODE plugin in Cytoscape, one module (MCODE score ≥11) was retrieved from the PPI network, and DEGs of the subnet were mainly associated with the cell cycle, cell division, and mitotic nuclear division (Figure 4E). The top ranked 10 hub DEGs were identified from the PPI network modules using the degree algorithm of the CytoHubba plugin as shown in Figure 4F, including *bub1*, *bub1b*, *kif2c*, NUF2 component of NDC80 kinetochore complex (*nuf2*), alpha-L-fucosidase 2 (*fuca2*), centromere protein H (*cenph*), *ccenpk*, trans-Golgi network protein 1 (*tgoln1*), fibrinogen alpha chain (*fga*), and apolipoprotein A1 (*apoa1*). These hub DEGs were mostly enriched in items regarding the cell cycle and innate immune response.

### Maternal HS Increased the mRNA Expression of Genes Enriched in the Cell Cycle of Fetal Intestine

**FIGURE 5:**
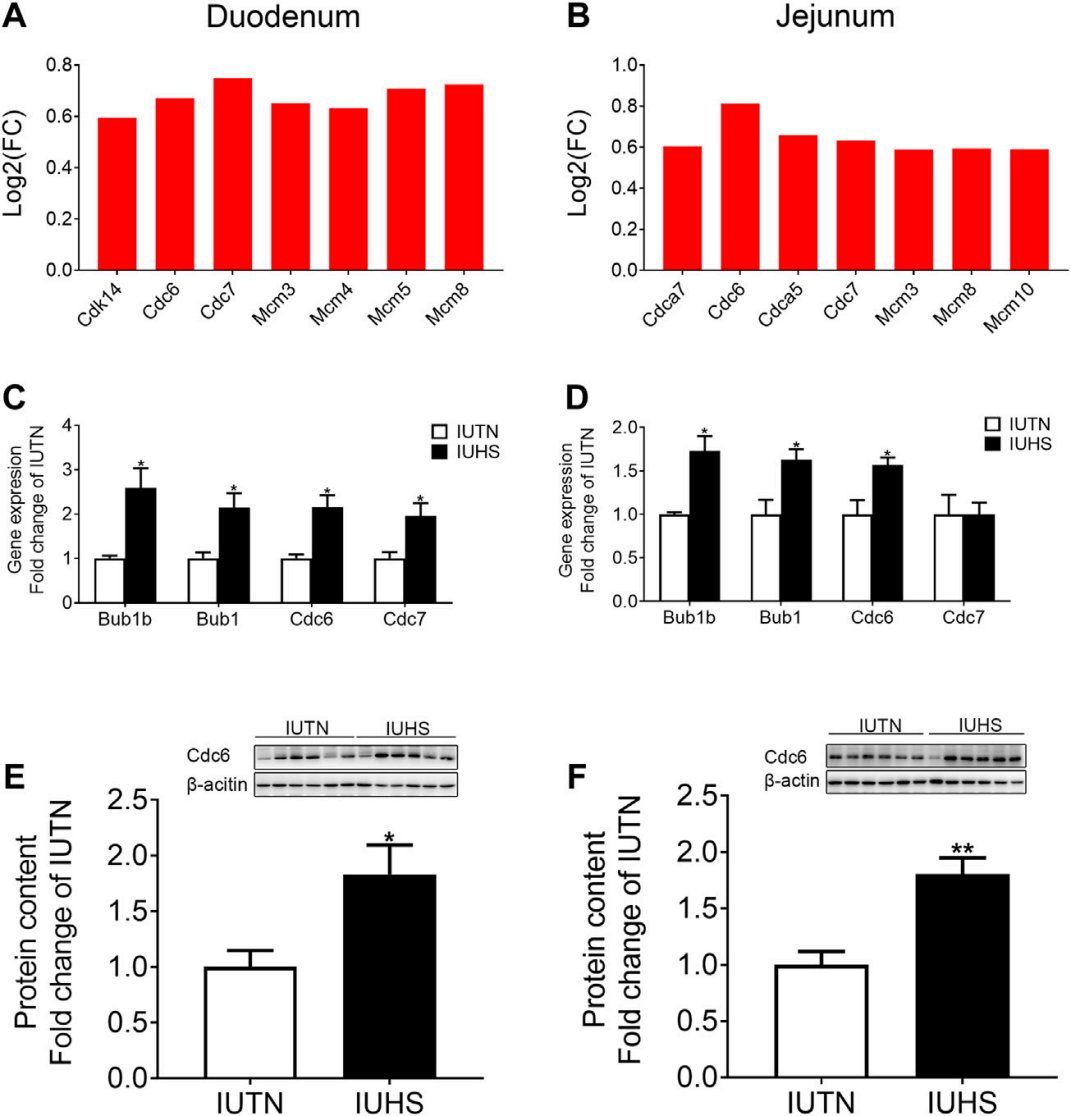
Effects of HS during late gestation on the cell cycle of fetal duodenum and jejunum. Fold change of DEGs **(A)**, mRNA expression **(C)**, and protein levels (IUTN: *n* = 8; IUHS: *n* = 5) **(E)** in the fetal duodenum. Fold change of DEGs **(B)**, mRNA expression **(D)**, and protein levels (IUTN: *n* = 8; IUHS: *n* = 5) **(F)** in the fetal jejunum. Gene symbols are shown on the x-axis, and log_2_ FC is shown on the y-axis. Red bars: up-regulation. Data are presented as mean ± SEM. *p < 0.05, **p < 0.01.

As shown in Figure 5A, compared with those in the IUTN group, the expressions of DEGs [cyclin-dependent kinase 14 (*cdk14*), cell division cycle 6 (*cdc6*), cell division cycle 7 (*cdc7*), minichromosome maintenance complex component 3 (*mcm3*), minichromosome maintenance complex component 4 (*mcm4*), minichromosome maintenance complex component 5 (*mcm5*), and minichromosome maintenance complex component 8 (*mcm8*)] associated with the cell cycle in the fetal duodenum were raised by maternal HS. For the fetal jejunum (Figure 5B), the expressions of genes [(cell division cycle associated 7 (*cdca7*), *cdc6*, cell division cycle associated 5 (*cdca5*), *cdc7*, *mcm3*, *mcm8*, and minichromosome maintenance complex component 10 (*mcm10*)] that were enriched in the cell cycle in the IUHS group were also up-regulated compared with those in the IUTN group.

To confirm the reliability of RNA-seq, the mRNA expressions of DEGs associated with the cell cycle were chosen for qRT-PCR in both the fetal duodenum and jejunum. For the fetal duodenum (Figure 5C), the mRNA expressions of *bub1b*, *bub1*, *cdc6*, and *cdc7* were up-regulated by maternal HS (*p* < 0.05). For the fetal jejunum (Figure 5D), maternal HS also raised the mRNA expression of *bub1b*, *bub1*, and *cdc6* (*p* < 0.05), but no significant difference was found in the mRNA expression of *cdc7* between the IUTN and IUHS groups. Additionally, the protein levels of Cdc6 in both the fetal duodenum (Figure 5E) and jejunum (Figure 5F) were increased in the IUHS group compared with the IUTN group (*p* < 0.05).

### Maternal HS Inhibited the Expression of Genes Enriched in the Intestinal Innate Immune System in Fetal Mice

**FIGURE 6:**
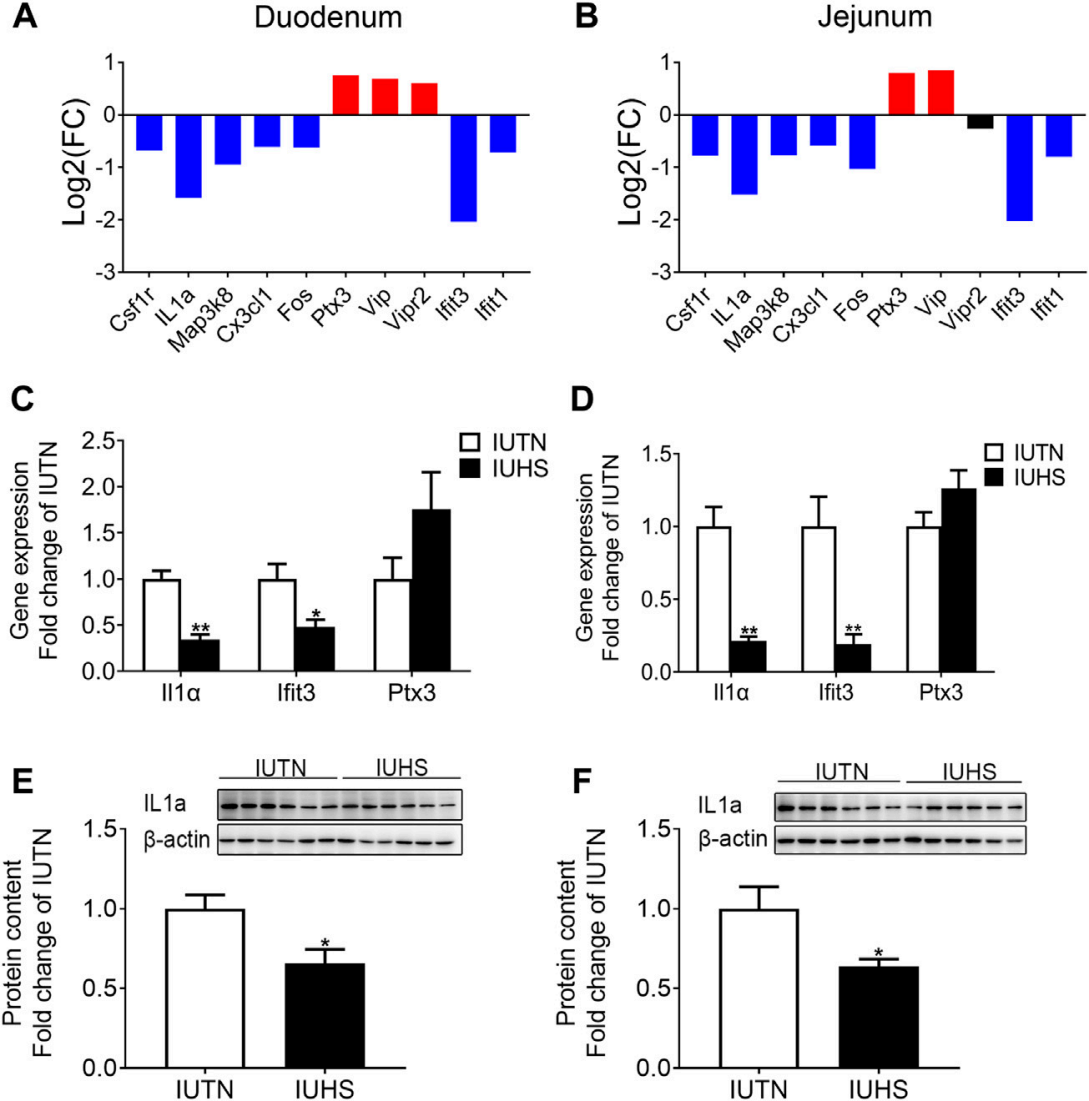
Effects of maternal HS on the innate immune development in the fetal duodenum and jejunum. Fold change of DEGs **(A)**, mRNA expression **(C)**, and protein levels (IUTN: *n* = 8; IUHS: *n* = 5) **(E)** in the fetal duodenum. Fold change of DEGs **(B)**, mRNA expression **(D)**, and protein levels (IUTN: *n* = 8; IUHS: *n* = 5) **(F)** in the fetal jejunum. Gene symbols are shown on the x-axis, and log_2_ FC is shown on the y-axis. Blue bars: down-regulation; red bars: up-regulation. Black: no difference. Data are presented as mean ± SEM. *p < 0.05, **p < 0.01.

For the fetal duodenum (Figure 6A) and jejunum (Figure 6B), the expressions of the colony-stimulating factor 1 receptor (*csf1r*), interleukin 1 alpha (*il1a*), mitogen-activated protein kinase kinase kinase 8 (*map3k8*), *cx2cl1*, fos proto-oncogene, AP-1 transcription factor subunit (*fos*), interferon-induced protein with tetratricopeptide repeats 3 (*ifit3*), and interferon-induced protein with tetratricopeptide repeats 1 (*ifit1*) were down-regulated in the IUHS group compared with the IUTN group, but the expressions of pentraxin 3 (*ptx3*) and vasoactive intestinal peptide (*vip*) were up-regulated in the IUHS group. Additionally, qRT-PCR was performed to validate the reliability of RNA-seq, which showed that the mRNA expressions of *il1a* and *ifit3* in both the fetal duodenum (Figure 6C) and jejunum (Figure 6D) were reduced by maternal HS compared with those in the IUTN group (*p* < 0.05). However, the mRNA expression of *ptx3* in the fetal duodenum and jejunum was no different between the IUTN and IUHS groups. The protein levels of Il1α in the fetal duodenum (Figure 6E) and jejunum (Figure 6F) were much lower in the IUHS group than those in the IUTN group (*p* < 0.05).

## Discussion

The prenatal environment has a direct effect on the gut during prenatal development (Jasarevic et al., 2018). Prenatal stress during pregnancy increased the risk of intestinal dysfunction in the offspring (Bale et al., 2010). However, the effects of HS during late gestation on the placental immune response and the development of fetal intestine and its innate immune system remain unclear. In the present study, we found that maternal HS altered the expression of genes involved in the placental immune response, as well as inhibiting the expression of genes related to the development of the intestine and its innate immune system.

We previously found that HS during late gestation increased the circulating maternal LPS corresponding to impaired maternal gut barrier integrity (He et al., 2021), and this may contribute to the alterations of the placental immune response. As shown by the results of the PPI network and hub genes, maternal HS had an obvious influence on the expression of genes enriched in the placental immune response, and these genes were up-regulated in the HS group compared with the TN group. Consistent with a previous report, maternal stress–induced inflammatory response in the placenta leads to the hyperactivity of the male mice (Bronson and Bale, 2014). However, it is necessary to study whether maternal HS causes the hyperactivity of the offspring in the future. The increase of macrophage marker CD68 mRNA expression caused the activation of complement response in the placenta, followed by the expression of complement-related genes *C1qa*, *C3*, and *CD55* being up-regulated, and induced the placental inflammation response (Teirilä et al., 2019). The expressions of genes including *adgre1*, cd68, *c1qa*, *c3*, and *cd55* in the placenta were raised by maternal HS. These data suggested that maternal HS might recruit the macrophage in the placenta, resulting in the activation of the complement system and immune response–related gene expression. Macrophages are of two types: pro-inflammatory activity (M1) and anti-inflammatory activity (M2) macrophages. Csf1 induced M2 macrophage differentiation (Jena et al., 2019). In the study, the mRNA and protein levels of placental Csf1 were decreased in the HS group compared with those in the TN group, indicating that maternal HS might reduce the numbers of M2 macrophages and the ability of anti-inflammatory in the placenta. However, these experiments remain to be performed in the future. Additionally, the concentrations of fetal immunoglobulins increased with the transfer of maternal immunoglobulin G (IgG) across the placenta during the third trimester of pregnancy (Sampah and Hackam, 2020). In the present study, the expressions of *fcγ1r*, *fcγ3r*, *ifi27*, and *ifi30* in the placenta were inhibited in the HS group compared with the TN group, which might be attributed to affecting the transfer of maternal IgG from the placenta to the fetus and decreasing the protective effect on the fetus. Ptgs2 also has anti-inflammatory effects (Mark et al., 2013). The mRNA expression of placental Ptgs2 was down-regulated by HS. We speculated that the inflammatory response might occur in the placenta under maternal HS conditions.

The alterations of maternal intestinal changes probably contribute to adverse maternal and placental adaptations, and via altering the development of the fetal gut (Gohir et al., 2019). We previously found that maternal HS disrupted the placenta barrier function, which might impair fetal development (Guo et al., 2021). The shorter villus height was observed in the IUHS group, indicating that maternal HS impaired the intestinal development and morphology in fetal mice. These results suggested that maternal HS might inhibit the capacity of intestinal nutrient absorption in the offspring. To further investigate the effects of maternal HS on the fetal small intestine, we used RNA-seq to explore the expression of genes associated with intestinal development in fetal mice. The data showed that HS during late gestation inhibited the expression of genes related to intestinal development in fetal mice. Fatty-acid–binding protein 2 (Fabp2) was a marker of intestinal development and reflected the cell renewal and maturation of the intestine (Reisinger et al., 2014). In the present study, we found that the expression of Fabp2 was down-regulated in the IUHS group compared with the IUTN group, indicating that maternal HS inhibited the cell renewal and maturation of the fetal intestine. The PPI network and hub gene analysis demonstrated that maternal HS had a profound influence on the cell cycle in the fetal duodenum and jejunum. Furthermore, the expressions of genes enriched in the cell cycle were raised by maternal HS compared with those in the IUTN group. A previous study reported that the cell cycle of intestinal epithelial cells increased before differentiation, which affected the maturation of crypt cells and slowed down the transfer rate of mature crypt cells to the villus (Srivillibhuthur et al., 2018). Therefore, we speculated that maternal HS inhibited the maturation of intestinal epithelial cells and slowed down the transfer of crypt cells in fetal mice. However, the underlying mechanisms of maternal HS inhibiting the intestinal development in the offspring remain to be investigated.

Our previous results found that HS during late gestation increased the intestinal permeability and induced intestinal barrier dysfunction of newborn piglets (Guo et al., 2020), which might affect the absorption of IgG and the development of innate immune function in the offspring. A recent study also reported that the immature gut barrier played an important role in establishing immunity in newborn mammals (Weström et al., 2020). Additionally, our data demonstrated that the expressions of *csf1r* and *Il1a* related to the intestinal innate immune system were down-regulated in the IUHS group compared with the IUTN group. Previous studies showed that csf1r deficiency in mice affects the Paneth cell genesis and normal intestine development (Huynh et al., 2009), regulates the release of inflammation mediators and is involved in the cellular inflammatory response (Schulze et al., 2008), possibly suggesting that maternal HS affected the establishment of the intestinal innate immune system in the offspring. In contrast to our results, maternal stress during pregnancy increased the expression and concentrations of intestinal pro-inflammatory cytokines and caused an inflammation response in the offspring (Jasarevic et al., 2018). Maternal stress during pregnancy also up-regulated the expression of intestinal complement C3 in the offspring, leading to tissue damage in a mesenteric ischemia model (Yeramilli et al., 2020). It is well known that the maturity of the mucosal immune system was different across species, for examples, in mice at 1 month (Levast et al., 2014), but in pigs and bovine at 6 months (Chase et al., 2008). These differences may be due to the differences in source, time, and intensity of the maternal stress. The immaturity of the innate and adaptive immune system in the fetal intestine stimulated the overgrowth of pathogenic bacteria, which caused the inflammatory response and increased the risk of infection (necrotizing enterocolitis) in the offspring (Weström et al., 2020). Therefore, we speculated that maternal HS decreased the ability of the fetal intestine to resist pathogenic bacteria, which in turn increased the risk of intestinal disease occurring in the offspring, and the relevant work will be performed in the future.

The present study has a potential limitation. As known, the previous study mainly focused on the effects of maternal HS on the placental barrier function and nutrient transport activity and metabolism (Guo et al., 2021; Silva et al., 2021; Zhao et al., 2021). In the present study, the expressions of genes enriched in the placental inflammation response were altered under maternal HS conditions, implying that maternal HS might cause the inflammation response in the placenta. To obtain the robust results, a variety of ways need to be performed to verify the inflammation response in the placenta, and the underlying mechanism is worth deep studying. In addition, it needs more research to study the long-term adverse effects of maternal HS on the offspring’s growth and development, including the alterations of microbial colonization and gut permeability.

## Conclusion

In summary, the results of the current study suggested that maternal HS increased the expression of genes related to placental immune response, which might contribute to the decrease of the intestinal villus height in the fetal mice. Additionally, the expression of genes related to fetal intestinal development and its innate immune system was also inhibited, which may be due to the increase of the expression of genes involved in the cell cycle and a longer renewal cycle of intestinal epithelial cells in the offspring.

## Data Availability

The datasets presented in this study can be found in online repositories. The names of the repository/repositories and accession number(s) can be found below: National Center for Biotechnology Information (NCBI) BioProject database under accession number GSE192968. The data were showed in the link: https://www.ncbi.nlm.nih.gov/geo/query/acc.cgi?acc=GSE192968.
